# Looking beyond values: The legitimacy of social perspectives, opinions and interests in science

**DOI:** 10.1007/s13194-022-00490-w

**Published:** 2022-10-04

**Authors:** Hannah Hilligardt

**Affiliations:** Leibniz Universität Hannover, Hannover, Germany

**Keywords:** Values in science, Iris Marion Young, Social legitimacy, Authority, Social value management ideal

## Abstract

This paper critically assesses the current debates in philosophy of science that focus on the concept of values. In these debates, it is often assumed that all relevant non-epistemic influences on scientific research can be described as values and, consequently, that science carries social legitimacy if the correct values play their proper role in research. I argue that values are *not* the only relevant non-epistemic influences on research: not unless our definition of values is so broad that it becomes unmanageable. Other factors also affect the authority and social legitimacy of science. I employ political theorist Iris Marion Young’s concepts of social perspectives, opinions and interests to attempt a differentiation of contextual influences relevant to scientific research. While problems arising from these influences may overlap, they often differ in important ways too. As a consequence, I argue that contextual influences cannot be managed jointly but require distinct and complementary strategies.

The legitimate role of values in science is one of the central topics in contemporary philosophy of science. As Bennett Holman and Torsten Wilholt (2022) put it, it is the “new demarcation problem” by which science and pseudo-science – or good and bad science – can be distinguished. For if we agree that (social) values play a necessary role in science,^1^ epistemic considerations are no longer sufficient to determine when science is ‘good’, that is: authoritative and trustworthy. Ethical and political criteria must be included, too.

This paper aims to contribute to this debate by critically assessing the conceptual framework on which it is based. Such a framework demands that all relevant non-epistemic influences on scientific research either *are*, can be related *to*, or can be conceptualised *as* values. Consequently, it is assumed that for science to carry social legitimacy, societal value influences must be managed appropriately. I argue that, unless our definition is very (and unmanageably) broad, values are *not* the only relevant non-epistemic influences on research. Other influences also affect social legitimacy and, thereby, authority of science.^2^ This has implications for the values in science debate. Differentiating between contextual influences helps better describe causal mechanisms and assess their legitimacy. For instance, the influence of vested interests should be judged differently than that of social experiences or background beliefs. Furthermore, if values are not the only relevant influence, the discussion on the “new demarcation problem” should shift its focus to extend beyond asking what the legitimate role of values in science is. Relevant questions could rather be: what are contextual influences on scientific research, what problematic effects can they have, and how does science have to be organised to prevent such effects?

To substantiate my argument, I will draw on the work of political theorist Iris Marion Young. Young also holds that values – understood as abstract criteria – are only one of various elements to consider in political decision-making. Consequently, she has developed a conceptual framework that aims to capture politically relevant aspects of people’s and groups’ identities that, according to her, should be represented in an inclusive democratic system: their *social perspectives*, *opinions,* and *interests*. I will adopt this framework to differentiate between contextual influences in science.

The paper will proceed in three steps. I begin with an account of how the concept of values is used in current debates in the philosophy of science. I point out some ambiguities and propose restricting the use of the term to the Kuhnian sense, that is, values as criteria of choice. In a second step, I discuss other contextual influences in scientific research that are not well described as values in the above sense. Lastly, I turn to the question of legitimacy. I discuss what it takes for science to carry social legitimacy and argue that such a requirement can only be fulfilled if a plurality of contextual influences is taken into account in their own right.

## Defining values in science

What do philosophers of science mean when they talk about values? One of the most commonly referred to conceptualisations can be found in Thomas Kuhn’s landmark paper “Objectivity, Value Judgement, and Theory Choice” (Kuhn, 1977). In this paper, he argues that the *criteria of choice* used in science to evaluate theories and choose between competing ones – accuracy, consistency, scope, simplicity, fruitfulness – should be understood and conceptualised as *values* (rather than rules, norms or maxims). As such, they have specific characteristics. Firstly, values are imprecise; they can be differently interpreted and applied. Secondly, values can conflict: a certain theory might be accurate but not widely applicable and thus necessitate certain trade-offs. As a result, values can never determine a choice or decision, but they do influence it and serve as grounds for justification. This does not mean that value disagreements are a mere matter of taste: they are subject to and perhaps necessary for rational debate. Finally, scientific values leave room for “rational men to disagree” (Kuhn, 1977, p. 332), which makes scientific pluralism, and (crucially, for Kuhn) scientific progress possible.

In the current debate, the values that Kuhn discusses (accuracy, scope etc.) are often referred to as *epistemic* or *cognitive* values and are contrasted with *non-epistemic* values. The influence of epistemic or cognitive values is uncontroversial and does not conflict with the traditional ideal of value-free science (cf. Brown, 2020; Büter, 2015; Douglas, 2016; Elliott, 2017; Hicks, 2014; Longino, 1996). Instead, the values in science debate asks what role *non-epistemic* values play in research, what values *ought* to be permitted to play a role, and what role they *ought* to play (cf. Büter, 2015; Holman & Wilholt, 2022; Rolin, 2021). In this context, values have gained a broad set of meanings. For instance, in *A Tapestry of Values*, Kevin Elliott broadly defines them as “something that is desirable or worthy of pursuit” (Elliott, 2017, p. 11). Shortly after, he says that “[v]alue judgements are scientific choices that cannot be decided solely by appealing to evidence and logic” (*ibid*., p. 12). On this account, the space opened up by rational disagreement is exclusively filled by values. Arguments that rely on or explicate this “gap”, like those that distinguish between epistemic and non-epistemic values, have in the past been criticised (Brown, 2013, 2020; Longino, 1996). Nevertheless, the assumption that *when* extra-scientific considerations and influences play a role, these can either be conceptualised as or traced back to values and value-judgements is shared by most strands within the values in science debate.

It is worth mentioning that Kuhn does not employ this line of reasoning. He points out that epistemic criteria of choice *are like* what we call values in a non-epistemic context, such as freedom of speech or quality of life (Kuhn, 1977, p. 330). He does not say that the aforementioned “gap”, the space that arises from the imprecise nature of the epistemic values, is closed by or filled with non-epistemic values. After all, he holds that values are characterised precisely by the fact that they leave a gap – and thereby leave room for disagreement. He does name various non-epistemic *factors* that might explain why scientists differ in their weighing and interpretation of the epistemic values at play: their past experiences, cultural background or cognitive disposition (e.g. how risk-averse they are) (Kuhn, 1977, p. 325). These factors are not values in the above sense, even if societal values might make up part of some of them (e.g. cultural background). This paper aims to draw attention to these other non-epistemic factors and the ways they influence scientific research.

In recent years, some philosophers of science have already drawn attention to existing ambiguities in the way the concept of values is employed in philosophy of science: In his book on *Science and the Moral Imagination*, Matthew Brown holds that the term ‘value’ is often used “as an empty placeholder, vaguely identified with ethics, political views, desires and wishes, or stakeholder interests” (Brown, 2020, p. 173). Justin Biddle has argued that what philosophers mean when they speak of “value free” science is that science “should be free from all contextual factors” (2013, p. 132). And Zina Ward (2021) has not only shown that what is considered a value is rather ambiguous but also that there are substantial differences in how philosophers of science characterise the relationship between ‘values’ and research. She writes that science is considered value-laden when values either “motivate, justify, cause, or [are] impacted by the choices we make” (*ibid*., p. 54). Yet, with the exception of Biddle, these thinkers argue that we should nevertheless stay within the conceptual framework of values and address existing ambiguities by differentiating more clearly between different values and value-influences.

Although I wish to retain the notion that a coherent account can be formulated this way, I will argue that labelling all contextual factors that influence scientific research ‘value-influences’ is unhelpful when it comes to formulating a new ideal for science-society interactions. In some cases, it might even be misleading, and for two reasons: Firstly, the value-terminology often implicitly relies on a dichotomy between facts and values, or between the normative and the descriptive. Various STS scholars have argued that these elements are often entangled to an extent where the very distinction becomes difficult to uphold (Jasanoff, 2004; Latour, 2004). Philosophers within the values in science debate have supported this claim by showing that scientific knowledge is not *solely* descriptive (Alexandrova, 2018; Büter, 2015; Intemann, 2020; Rolin, 2021), and, conversely, that moral judgements require empirical input (Anderson, 2004). Yet, in ongoing attempts to identify ‘values’ in science, philosophers tend to slip back into the dichotomy that underlies the traditional ideal. This is because attempts to separate values from evidence and logic quickly turn into an exercise of separating those judgements about which there can be reasonable disagreement from the things we need to accept, that is to say: an exercise of separating values and facts. Using different concepts can be helpful to consider science-society interactions from a different angle and to better bring different issues into view. Secondly, when contextual influences on science are subsumed under the header of ‘values’ this might suggest that they can all be managed in the same manner, or that a single strategy might be found that demarcates legitimate from illegitimate value influences. I argue that this is not the case; instead, different factors must be considered and managed differently.

My approach therefore differentiates between several contextual factors in science and proposes reserving the concept of “values” for the Kuhnian sense. To make this differentiation productive, I draw on the work of Iris Marion Young, a feminist political theorist. In her 2000 book *Inclusion and Democracy*, she argues that neither the representation of diverse values nor the formal inclusion of citizens in decision-making processes that pertain to values can guarantee democratic legitimacy. In an inclusive democracy, she says, citizens’ *social perspectives*, *opinions* (which include Kuhnian values), and *interests* must be represented in relevant decision-making institutions and public discourse. Starting from her account, I propose that contextual influences on scientific research are better understood when viewed through these three concepts than when unified under the rubric of ‘values’. In the following three sections, I will discuss Young’s suggestions and their respective roles in science.

## Social perspectives in science

The first concept I turn to is that of a social perspective. What Young calls social perspective has been discussed in philosophy of science in the context of feminist standpoint theory and the situated knowledge thesis. In this sense, her account is not new to the debate. Yet I argue that this concept allows us to group some contextual influences that are important in the values in science debate, such as the role of social knowledge as evidence and blind spots resulting from a lack of specific experiences, and to designate these influences more accurately than ‘values influences’.

The experiences any individual has depend, to a large extent, on their specific position in a society. Such positions therefore produce socially embedded knowledge and a particular perspective for evaluation and orientation. Young calls this situatedness people’s “social perspectives”. Demographic markers such as gender, ethnicity, class, ability, and sexuality significantly influence a person’s perspective and play a distinctive role as criteria when it comes to the representation of perspectives. But perspectives can also arise from activities and decisions taken later in life, such as a person’s profession or family life. Perspectives influence the starting points and questions raised by an individual or a group, the effort and time required to understand somebody else’s experience, and the attention given to different issues. But they do not determine what outcomes somebody advocates for and what interests and values they pursue. As Young says: the “[s]ocial perspective consists in a set of questions, kinds of experience, and assumptions with which reasoning begins, rather than the conclusions drawn” (Young, 2000, 137).

Arguably, the representation of social perspectives in politics^3^ is especially significant in cases where problems are new, complex or ill-defined or, as Jane Mansbridge (1999, 2015) puts it, where interests are “uncrystallised”. Mansbridge and Young both cite the example of sexual harassment allegations at a time when the concept had not yet been incorporated into the political and legal framework: Women legislators, in many of these cases, were more prone to follow up on allegations and take them seriously than their male colleagues, irrespective of their political positions (Mansbridge, 1999, p. 647; Young, 2000, p. 140). This can be explained if we assume that sharing certain experiences makes it easier to understand and take seriously testimonies that, as Miranda Fricker (2007) has described it, lack clarity due to hermeneutical lacunae or that are subject to testimonial injustice. This is not an automatic process; not all women take other women seriously (cf. the discussion on this in Manne, 2018, p. 263), nor do all women share the relevant experiences. But this does not take away from the point that social perspectives *can* be important to develop new hermeneutical resources, shape political opinions, and counter testimonial injustices. As science is often concerned with new and complex problems and developing concepts to better represent real-world processes, one may expect this to hold in research, too.

As mentioned, Young’s account of social perspectives resembles some formulations of standpoint theory in feminist philosophy of science—and not by chance. Young adopted the situated knowledge thesis from Donna Haraway (Young, 2000, p. 114) and stood in conversation with other standpoint theorists like Nancy Hartsock (Hartsock, 1983, p. 306). But there are differences too. First of all, Young distances herself from some formulations of the thesis of epistemic privilege. She argues that members of structurally disadvantaged groups are just as liable to bias and misjudgements. Nevertheless, less privileged people are likelier to perceive and point out *dominant* biases and partialities (Young, 2000, p. 117). Secondly, Young does not have to concern herself with the question of what political education or critical reflection is necessary for a perspective to turn into an epistemically beneficial standpoint (Hartsock, 1983, p. 285; Harding, 1986, p. 26) because she conceptually separates perspectives from political viewpoints (discussed in the next section). There are several advantages to this separation. Feminist philosophers of science have debated whether women or feminists brought about the rethinking of many androcentric or sexist presuppositions in many scientific disciplines (Wylie, 2012, 64ff.; Kourany, 2010, 64ff.). From Young’s perspective, we can say that both influences play a role that is at least analytically separate. For instance, social perspectives (those arising from a person’s gender) provide a researcher with specific experiences that can be epistemically and politically beneficial; political commitments and education can serve other purposes and come with other pitfalls. Social perspectives are hence not standpoints because they do not presuppose critical engagement.

One contextual influence on scientific research that can be better understood as part of people’s social perspective rather than as a value influence is *experience* serving as evidence. Standpoint theorists have discussed this type of influence since the 1980s, particularly emphasising the epistemic benefits of the experiences of marginalised group members (cf. Intemann, 2010; Wylie, 2012). They argue that the systematic lack of marginalised perspectives, with their experiences, within the scientific community can lead to blind spots with both epistemic and ethically problematic consequences. A case in point is Anne Fausto-Sterling’s classic example of research on differences in visual-spatial capacities between men and women (Fausto-Sterling, 1985, pp. 30ff.). Particularly relevant is her discussion of the so-called rod and frame test, an experiment in which participants were given the task of aligning a rod to the room, ignoring a tilted frame around it (or their tilted chair). In most experiments Fausto-Sterling is concerned with, male participants performed slightly better than female participants; in no repetition, women performed better than men. It was concluded that biologically speaking, men have better visual-spatial capacities than women, a notion that stubbornly persists in folk wisdom. But most scientists conducting the experiments did not consider that female participants might have felt uncomfortable in a dark room with a male researcher or that women were and often still are socialised differently. As Kristen Intemann puts it:

White middle-class and upper-class researchers in the 1960s were unlikely to have had the experiences that would make them aware of what it was like to be nervous about being in a dark room with a strange man or to be discouraged or penalized for being assertive or demanding. As a result, *it probably never even occurred to them* that the experiment design might hinder the performance of females. (Intemann, 2009, p. 258; emphasis added)

Versions of the experiment that did take these factors into account found no significant differences in performance (Fausto-Sterling, 1985, p. 32).

The issue of blind spots is one important example of why social perspectives (and the lack thereof) matter in science, but it is often treated as a case of value-laden science. In a paper on values in science, Intemann herself labels the rod and frame tests a case of “value judgements” influencing scientific research (Intemann, 2020, p. 207). Yet even if the result of such influences can (under some definitions) be called value-laden, it is unhelpful to equate social perspectives with ‘values’. The failure of male researchers in the 1960s to understand what effect the experimental set-up of the rod and frame test would have on those socialised to behave womanly is not in itself sexist and not per se based on a judgement of how women should behave. When a thought or implication does not occur to somebody, it often demands an external influence for them to be made aware of its possibility. But when somebody actively insists on the correctness of their position or pursues conflicting, non-epistemic aims with their research, being made aware of other perspectives often has no effect. Blind spots therefore demand different measures than openly acknowledged value disagreements. I will return to this point in the final section.

## Opinions in Science

The second contextual influence often subsumed under discussions of value influences is background assumptions or beliefs. I consider them ‘subsumed’ because background assumptions are not always treated as *values*. But they are often considered either value-laden (i.e. normative) or are themselves assumed to be influenced by values. The focus is consequently on values as relevant influences. In the political domain, it is more common to treat background assumptions and normative beliefs as holistic phenomena (Young terms them *opinions)*. I propose to do the same within philosophy of science. I will begin this section by presenting Young’s conceptualisation of opinions and then relate this to Helen Longino’s account of background assumptions in science.

Using the term opinion in the context of science can be misleading: there is a connotation of opinions as unfounded, personal views that should, many would contend, be kept out of science. Young’s use of the concept, however, is more nuanced. She defines opinions as “the principles, values, and priorities held by a person as these bear on and condition his or her judgement about what policies should be pursued and ends sought” (Young, 2000, p. 135). This can include ideologies or worldviews (such as feminism, Marxism or neoliberalism), religious beliefs, and cultural traditions. For Young, the main channel of representation for opinions in contemporary Western representative democracies is through political parties. A ‘green’ party or a ‘conservative’ party, for instance, represents a certain set of opinions: that the environment should be protected, that immigration should be restricted, that governments should interfere as little as possible in the market, and so forth. Furthermore, these opinions are prioritised: members of a green party likely consider environmental concerns more pressing than questions around cybercrime. Young holds opinions can be contested and more or less well-grounded, though it remains a hallmark of democracy that pluralism of opinions must be granted.

Two things should be noted about this conceptualisation. Firstly, Young is vague about her intended scope of what she calls ‘opinion’. In everyday language, we often use the term to describe isolated standpoints on issues at hand, and at some points, Young employs this use too. However, for this paper, it is helpful to think of opinions as holistic, comprehensive phenomena, similar to what we might call systems of belief or world views. In support of such reading, Young writes:

While I doubt that most people’s opinions on public matters all derive from a single ‘comprehensive doctrine’, I do assume that most people make judgements about particular social and political issues with the guidance of some values, priorities, or principles that they apply more broadly than that case, if not to all cases. (Young, 2000, p. 135)

The focus then lies on higher-level, organised opinions such as feminism or neo-liberalism. Secondly, although there is an emphasis on “what policies should be pursued and ends sought” in Young’s characterisation of opinions, it involves both normative and descriptive elements. Shortly after she writes that “[b]y opinion, I mean any judgement or belief about how things *are or ought to be*, and the political judgements that follow from these judgements or beliefs” (*ibid*., emphasis added). I want to illustrate this last point using an example from climate science.

As journalist Eric Conway and historian of science Naomi Oreskes have documented (Oreskes & Conway, 2010), the doubt-mongering campaigns on the reality and anthropogenic nature of global warming involved a good number of scientists who were strongly (and problematically) influenced by what with Young we can call their own political opinion (Fred Singer, Frederick Seitz, Bill Nierenberg and Robert Jastrow, among others). The opinion these scientists defended *both* involved values (primarily freedom) *and* a set of beliefs and assumptions that made it a comprehensive worldview or ideology to begin with. Arguably these beliefs, although descriptive in nature, are equally or even more important to understand than the values they defended. As Conway and Oreskes describe, Singer, Seitz, Nierenberg and Jastrow were deeply committed to the fight against communism. They believed government regulation, including environmental regulations, to be a slippery slope into communism and totalitarianism. In their latest book, *The Magic of the Market Place* (Oreskes & Conway, 2022), Oreskes and Conway trace back the emergence and rise of this narrative. They argue that American corporations throughout the second half of the twentieth century went through quite some pain to popularise and back up the slippery slope argument by marketing and simplifying, among other works, economist Friedrich Hayek’s book *The Road to Serfdom.* The result was the popularisation of the idea that freedom *necessarily* involves representative democracy, political freedom and *free enterprise.* When one of the three is compromised, the whole structure collapses. This slippery slope narrative is a good example of an opinion's descriptive element. It is neither a value nor normative, yet has influenced the scientists’ work. This sense is captured when we speak of ‘opinions’ influencing science rather than ‘values’.

The descriptive elements of opinions explain why, unlike social perspectives, some opinions can be “more well-founded than others” (Young, 2000, p. 135). For instance, in contemporary politics, opinions differ sharply with regard to the question if climate change is real and anthropogenic or if Covid-19 exists and vaccines provide some protection against infection. On questions such as these, empirical research supports one opinion significantly better than the other.^4^ However, other background assumptions are ontological, theological or metaphysical in nature and cannot straightforwardly be proven right or wrong by science. We speak, for instance, of ethicists being moral realists or philosophers of language being pragmatists. Both positions involve descriptive beliefs concerning how the world is; but there can be reasonable disagreement about these beliefs. Young characterises opinions as a combination of such different elements.

Within the philosophy of science, Helen Longino is a thinker who has drawn attention to the importance of background assumptions. Consider the following example: in *Science as Social Knowledge* (Longino, 1990), Longino engages in a self-reflective discussion of the influence of her feminist commitments on her work on the biological basis of gender differences. She specifically discusses a paper she wrote together with Ruth Doell (Doell & Longino, 1988) in which they argued against a “linear-analytic model” of the relationship between sex hormones and later behaviour and for a “complex model” of said relation.^5^ In *Science as Social Knowledge*, Longino argues that their interpretation and assessment of relevance of the available data depended in part on the model they chose. Evidence could therefore not conclusively decide which model is right. This underdetermination argument is by now familiar in the values and science debate. Yet what is interesting is that Longino does not always speak of ‘values’ only, filling the gap between evidence and theory. In this example, for instance, she writes:

Our [Doell’s and Longino’s] *political commitments* […] presuppose a particular understanding of human action, so that when faced with a conflict between these commitments and a particular model of brain-behavior relationships we allow the political commitments to guide the choice. (Longino, 1990, p. 190f; emphasis added)

In her description, a political commitment is comparable to Young’s concept of an opinion, as it involves both normative and descriptive elements. She delineates *background assumptions* concerning human agency, which, as she says, do not “contain normative terms” themselves (*ibid*., p. 190). “Values” enter the process because the decision to adopt certain background assumptions rather than others is motivated “by the desire to understand ourselves and others as self-determining (at least some of the time)” (*ibid*.).

When we consider how Longino describes the importance of political commitments in science, this type of influence seems better described by Young’s concept of an opinion than as “feminist values”. The normative and descriptive dimensions are often closely entangled, and citing abstract values such as equality or freedom is less informative than making transparent the (descriptive) background assumptions one is operating with. The consequences of this differentiation for the legitimacy debate will be discussed in the final section of this paper. First, I will look at Young’s third and potentially most challenging concept, interests.

## Interests in Science

Distinguishing between interests, opinions and values is challenging given that the concepts can be, and are, applied and defined in very different ways. And there often is overlap: if we understand a value construed broadly as “something that is desirable or worthy of pursuit” (Elliott, 2017, p. 11) and to value something as “to be disposed to act for the sake of that thing” (Brown, 2020, p. 115) interests can be understood as values. Heather Douglas, for instance, envisions her 2009 book *Science Policy and the Value-Free Ideal* as answering the following questions:

Which interests are relevant and why? Why is the exposure of interests important to the integrity of science? How does this fit with the ideal of value-free science, in which one’s interests are not to interfere with the interpretation of evidence? (Douglas, 2009, p. 19)

Note that *value*-freedom is defined here as the absence of direct influences of *interests*. For her purposes, values and interests appear indistinguishable or not worth distinguishing. Dan Hicks has a similar position. In a paper on the legitimacy problem (Hicks, 2014), they discuss two case studies for “value influences” in science: feminist values in archaeology that led to the challenging of sexist biases in the field and commercial values in pharmaceutical research that led to “the publication of false and misleading evidence” (*ibid*., p. 3279). While I agree that value judgements played a role in both cases, one might wonder if the influence of pharmaceutical companies is best framed as an influence of commercial ‘values’: would we not generally speak of a company’s *interests*?

To differentiate between values and interests, I focus on two important aspects of Young’s definition of interests. Firstly, she writes of interests as “what affects or is important to the life prospects of individuals, or the goal of organizations” (Young, 2000, p. 134). This means they are both instrumental to and dependent on the goals that one aims to achieve:

I define interest here as self-referring, and as different from ideas, principles, and values. The latter may help define the ends a person sets for herself, where the interest defines the means for achieving those ends. (*ibid*.)

On this definition, when we imagine a group that believes global warming is a problem that needs addressing, it is in that group’s interest to gain power, attention and influence and reduce the power and influence of fossil fuel companies. Now, while this characterisation works for some cases, it does have drawbacks. The instrumental goods typically considered to be in a person or group’s interest – money, power, prestige – seem to be the ‘goals’ of many. And while we might, in turn, give reasons for why these goods serve other purposes, differentiation is difficult when the same things can be means and ends.

Young’s second characteristic of interests, then, is perhaps more helpful. She claims that what separates interests from opinions is that the former are self-referring and not generalisable. It is not obvious what Young might mean by employing the term “self-referring” given that a person can very well represent another person’s interests. But what seems to be crucial is that when we speak of interests, we are referring to *somebody’s* interests. Interests can be shared; some are shared by a public, even a global public. But the group or public with such shared interests must be a defined collective within which individual interests are, and have to be, weighed against each other. One difference between considering opinions and interests is then that thinking in terms of interests forces us to focus on specific agents, their goals and their relations to other agents.

Take the following example: Naomi Oreskes’ *Science on a Mission* (2020) describes the effect of Navy funding on post-war oceanography. During the Cold War, the possibility of submarine warfare rendered knowledge of the deep sea, particularly physical oceanography and marine geophysics, highly valuable to the US army. The Navy became the main patron of American oceanographic research and, as European oceanography was severely damaged after WWII, a leading influence on global research of the field (*ibid*., p. 497). This influence, although not opposed to epistemic research goals, had a profound impact and caused some questions to largely disappear from the radar. Marine biology was one of the fields that had a hard time winning grants because it was of little interest to naval warfare. As a consequence, “basic questions about fish and fisheries remained unanswered. Scientists [at the end of the twentieth century] literally did not know how many fish there were in the sea” (*ibid*., p. 496). Given the current collapse of global fish stocks and the role of marine life in stabilising marine ecosystems, this lack of knowledge from today’s perspective is highly problematic.

Military funding is a particularly good example of the influence of interests because in this case the ideological commitments of researchers, their beliefs and their values only partially explain why post-war oceanography researched currents rather than fish.^6^ The focus on “the ocean as physical medium through which sound and submarines might travel” (*ibid*., p. 497) can be understood better knowing what the Navy paid *for* – what its interest was when it financed this field of science. Paying attention to the influence of interests in science is especially important because decisions that benefit powerful groups in society are often justified with reference to values and the general good. As touched upon above, the tobacco and fossil fuel industries, for instance, rather successfully justified economic measures that served their interest with reference to the values such as freedom and individual choice (Oreskes & Conway, 2010, 2022). An answer to the question *cui bono?* must therefore find some space within a conceptual framework used to assess the legitimacy of contextual influences in science.

In sum, Young’s adapted framework involves three elements: social perspectives, opinions and interests. All three are often discussed as values in philosophy of science. In contrast, I have restricted the meaning of values to a Kuhnian sense; as such they are part of Youngian opinions. The main differences between the four concepts as used in this paper can be summarised as follows:

**Table:** 

	**Social perspectives**	**Opinions**	**Values**	**Interests**
*Definition*	An individual’s or group’s position in society and the experiences and knowledge that come therewith	A set of beliefs, values and principles that bear on a person’s judgements, goals and prioritisations	Abstract criteria of choice that can guide and justify actions. Values are an aspect of opinions	The means that are necessary to achieve the goals of individuals, groups and organisations. Interests are always the interests *of someone*, they can be shared but not generalised
*Example*	Women’s perspective, workers’ perspective…	Feminism, Marxism, Neo-liberalism…	Freedom, security, justice…	Power, money, influence, physical well-being…

## Managing contextual influences in science

I began this paper claiming that the legitimacy debate’s framework should be critically assessed. I hope to have substantiated this claim by showing that not all relevant non-epistemic influences on scientific research are values and that we can benefit from increased clarity and precision when we differentiate contextual influences. I now turn to the impact such differentiation has on our account of “the demarcation problem” to argue that science’s social legitimacy and credibility depend on more than the correct values playing their proper role in science. Differing contextual influences demand different strategies to manage them appropriately.

Holman and Wilholt (2022) find the main concern motivating inquiries into a new ideal of science-society interactions to be the following: if scientists’ values influence their work, reasons have to be given why those who have other values should believe the results of their research. The value-free ideal was meant to prevent this, guaranteeing science’s “veracity, universality and authority” (*ibid*.). The new ideal should fulfil the same function: it should prevent values from unduly influencing scientific research, as well as provide criteria to judge when influences are legitimate from a political and an epistemic point of view.^,^^7^,^8^ I believe that this is indeed an urgent task for philosophers of science. Take the ideal of authority: according to Holman and Wilholt, science has authority when it produces “a trustworthy body of knowledge that has broadly recognised social legitimacy” (*ibid*.).^9^ In turn, this allows scientific research to act as a “transpolitical” institution, or as Andrew Schroeder has put it, to serve “as a premise in practical reasoning” (2021, p. 553). If social legitimacy is low, this poses severe problems in those areas of policymaking where we need to rely on science to navigate the interactions between human and nonhuman actors, such as in the Covid-19 pandemic or climate change.

Yet if this is the aim, then a framework where all non-epistemic influences are conceptualised as values is not conducive to solving the issue at hand. Based on Young’s account of democratic decision making, I argue that the social legitimacy of science depends on more than the appropriate management of values alone. To show this, the following part will look at potential problems that occur when social perspectives, opinions, and interests influence scientific research, after which it discusses how these various influences could be managed to prevent negative effects. This sketch will necessarily be incomplete; there are many ways in which contextual influences can come to bear on science. It is primarily intended to illustrate the benefits of adopting Young’s conceptual framework to make relevant differences visible.

### Managing social perspectives in science

We remember that social perspectives provide a person with knowledge and experiences and prompt certain questions to be of relevance to certain people rather than others. Social perspectives in themselves cannot be illegitimate or wrong (Young, 2000, p. 147), but it can be problematic when a group is composed of members with a homogenous social perspective, as this can lead to blind spots and a lack of relevant knowledge. An example I gave for such phenomena was the research on spatial abilities; in this sphere, explanations for women’s lower performance that stem from socialisation and the experimental set-up simply did not occur to most “[w]hite middle-class and upper-class researchers in the 1960s” (Intemann, 2009, p. 258). Measures to appropriately manage these researchers’ values would not be sufficient to legitimise their work because their community lacked the necessary social diversity to prevent problematic blind spots. Proposals to remedy this specific problem include promoting members of underrepresented groups, removing structural barriers that prevent members of such groups to work in a field, and so forth (Intemann, 2009; Rolin, 2020). Where people with a relevant perspective cannot or do not want to be included in the scientific community, dialogue with them should be sought (Brown, 2008; Wylie, 2015). Obvious examples here are patient groups, or indigenous groups that might have relevant knowledge on certain experiences, areas, or ecosystems, or have certain relevant skills. The crux of managing social perspectives is not how to exclude them, but rather how to create diverse communities and determine which perspectives are relevant to an issue at hand.

### Managing opinions in science

With regard to the influence of opinions, it seems that other worries and potential problems are prevalent, and other measures are suited to address them. Usually, opinions are represented in a broadly defined political position such as feminism or conservatism. From an epistemic perspective, one might worry that such political or religious opinions are sometimes held dogmatically, which would constitute a concerning influence on scientific research. Elizabeth Anderson writes that “the worry [of defenders of the value-free ideal] is that if we allow value judgments to guide scientific practice, they will infect it with dogmatism, thereby rendering it blind to the evidence” (Anderson, 2004, p. 3). Matthew Brown raises the same concern, although he specifies that this pertains not to worldviews in general but what he calls “ideologies” only:

I will reserve the term *ideology* to refer to a certain kind of worldview that is problematic in structure. Like any worldview, an ideology is a complex evaluative standpoint that combines both evaluative and factual commitments. These commitments are unified into a kind of self-reinforcing structure that allows all new evidence and experience to be assimilated to the ideology. The end result is closed-mindedness, with few resources from the inside that permit critique of the ideology. (Brown, 2020, p. 142)

If we accept that this is a possible but not inevitable characteristic of certain types of opinion and that dogmatism is harmful to the epistemic goals of scientific research, then the opinions that influence science must be managed in a way that prevents dogmatism.^10^ Again, note that the worries about dogmatism do not solely emanate from values: the ‘factual’ elements of scientists’ opinions may lead to biases as well. The legitimacy of science depends not just on proper value management, but on proper management of background beliefs, too.

Fostering a pluralism of opinions is one possible approach to achieve this aim, as Helen Longino has argued (1990).^11^ When entering a dialogue with representatives of opposing opinions, underlying background assumptions can be laid open and the dogmatic holding on to a view in spite of strong counterarguments and contradicting evidence can be exposed. Importantly, however, different measures are necessary to achieve such political diversity than to achieve *demographic* diversity. Kristina Rolin (2016, 2021) has pointed out that existing relations of power can make it difficult for certain opinions to be challenged (see also Schönwitz, 2022). To counter the effects of these relations of power so-called scientific/intellectual movements can play an important role to offer support, empowerment and constructive feedback rather than outright rejection of ideas that challenge a dominant view. The creation of such structures goes beyond increasing the social diversity of the scientific community, even though they might sometimes reinforce each other.

To argue for pluralism of opinions does not exclude the possibility that certain politically or scientifically illegitimate opinions are barred from discussions. Indeed, the establishment of a communities’ core values (in a Kuhnian sense) can serve such a purpose. If scientists or scientific institutions agree for instance that non-discrimination is a core value of their community, racist opinions can be deemed illegitimate and excluded. Who is to set those boundaries, who is to enforce them and on what grounds remains an open question (cf. Leuschner, 2012; Biddle & Leuschner, 2015; Melo-Martín & Intemann, 2014). But the differentiation between opinions and values is helpful for these debates for the way it draws attention to a problem with “right value” approaches^12^ such as that of Janet Kourany. Kourany proposes that scientific research should be based on the right values, namely egalitarian ones (2010). She goes on to claim that egalitarian values are the values that are shared in and motivate feminist research (*ibid*., p. 76). But there is a significant gap between a scientific community that acknowledges equality as one of its core values (and therefore excludes openly discriminatory research) and feminist research, the latter comprising a host of background assumptions and political commitments. There can, and I claim there should be, a diversity of *opinions* in science: this does not preclude agreement on certain core values that provide general guidance and help determine the limits of what is deemed a legitimate opinion. The social legitimacy of research is dependent on the appropriate management of both factors.

### Managing interests in science

Lastly, I turn to interests and the question of how their influence on science is to be managed appropriately. Two problems appear particularly pertinent here. Firstly, when the private interests of researchers (or patrons) conflict with epistemic aims, this can prompt biases or even manipulation of research. In medical research, this has been traditionally described as a mismatch between the primary interests (professional duties, patients’ health etc.) and secondary interests of researchers or practitioners (financial gains, prestige etc.) (cf. Thompson, 2017). One way to prevent such conflicts of interest is to create an incentive system within science that aligns primary and secondary interests. Alternatively (or additionally), conflicts of interests can be made transparent, checked for and excluded, for instance by journals (cf. Michaels, 2008 on some efforts taken by journals to prevent problematic influences of industry interests).

The second problem is that the various members and groups of a society have vastly unequal opportunities to influence research in a way that serves their interests. Such societal conflicts of interests are structurally different from conflicts in opinion as they do not concern what a person believes (or believes to be right or wrong). We can agree on societal goals but disagree on who should bear their costs and risks. Similarly, we might agree that health or security are valuable, but disagree on whose health and whose security should be prioritised. A philosophical account that is concerned with the issue of balancing different interests is Philip Kitcher’s model of well-ordered science.^13^ He provides a deliberative ideal in which all interests are represented and weighed appropriately (2001, 2011). Such a hypothetical scenario (potentially actualised in form of mini-publics [Kitcher, 2011, p. 130; Fishkin, 2009]), could serve to inform researchers’ judgements so that they are in line with “the common good” and no individual interests get to dominate science.^14^ My aim at this point is not to evaluate if mini-publics are a suited measure to the task. Instead, I contend that the social legitimacy of science will always depend in part on how the influence of interests is managed, in addition to the way in which opinions and social perspectives (and other potential influences not covered here) are managed.

To draw this section to a close, allow me to point out and respond to a potential objection to Young’s conceptual framework. As she herself admits, social perspectives, opinions and interests are in practice often represented in mixed forms (Young, 2000, p. 133). Furthermore, certain demographic markers are often used as proxies for one another in cases where they statistically correlate. It has been argued, for instance, that a diversity of social perspectives, on average, brings with it cognitive diversity and diversity of opinions (Rolin, 2020). Likewise, it is a common assumption that if a representative is a member of a certain social group they will be a better representative of that group’s interests as well (Mansbridge, 1999, 2015). Lastly, there is often a significant overlap or relation between opinions and interests. Think of the efforts industries have made to promote a “free-market fundamentalism” that served their commercial interests (Oreskes & Conway, 2022). In the face of these interactions and correlations, it seems that Young’s differentiation easily collapses and thereby loses practical use.

Young herself insists that this is not so. Social perspectives, in her account, shape and limit what views and opinions a person might have. They also influence one’s interests; a man for instance is differently affected by patriarchal systems than a woman and will therefore more likely have an interest in upholding it. But – and this is important for Young as she is responding to criticisms that have been raised against identity-politics approaches – a social perspective determines neither opinion nor interest (Young, 2000, Chapter 3). Among other reasons, this is because social experiences are so complex that individuals who share a similar background can emphasise different elements of their identity, set different goals and reason differently from their experiences (*ibid*., p. 137). To equate social perspectives, opinions and interests can therefore quickly lead to an essentialisation that takes away from people’s capacity to determine their own life goals. And there are practical reasons, too, for why it is problematic to uncritically assume that one marker of identity can be used as a proxy for another: Patricia Hill Collins has pointed out that the inclusion of underrepresented groups in systems marked by historic inequalities – such as the academic system – often does little to increase the diversity of opinions or balances interests. Instead, a few “safe” members of marginalised groups who support dominant beliefs get accepted into the ranks of academia. They then serve to legitimise the system while the underlying power structures remain unchallenged (Hill Collins, 2000, p. 254).^15^ Young’s pluralist proposal is an attempt to challenge this problem and give space to the importance of social situatedness for any form of inquiry or decision-making process in a way that is neither essentialist nor socially deterministic.

## Conclusion

The approach of this paper is meant to provide an alternative perspective to current discussions of science-society interactions in the values in science debate and the question how to manage various contextual influences on scientific research. It should not be understood as a comprehensive account of the ways in which research and politics, or societal debates more generally, relate. Other influences might play a role as well and, depending on the issue at hand, other conceptualisations might be more fruitful. Emotions, for instance, can be highly relevant and find little space in the three-part framework that I have adopted from Young (cf. Anderson, 2004; Roeser, 2018). Furthermore, it might be argued that framing science-society interactions in terms of (seemingly) external influences on science is also incomplete. For as the term “interactions” suggests, this is a two-way relationship, not one side influencing the other (Anderson, 2004). Lastly, readers might find the three concepts I have proposed – social perspectives, opinions and interests – too broad, vague or interdependent to serve demarcation purposes. The reason why I nevertheless present and defend this framework as helpful is that it draws attention to the fact that decision-making, within and outside of science, is rarely determined by moral/ethical considerations alone. Beliefs about what is right and what is wrong are not the only factor that drive people and not the only relevant point of conflict and disagreement between groups. This holds for the political domain as much as for science.

The trade-off (or advantage, depending on your point of view) of this approach is that any evaluation of the social legitimacy of a specific case of scientific research will take a gradual, rather than binary form (i.e. legitimate/illegitimate). It is imaginable, for instance, that a research community has mechanisms in place that successfully prevent the direct influence of interests but is very homogenous in terms of its social perspectives and opinions. In such a case, we can say that its claims carry *more* social legitimacy than claims made by scientists who have or represent strong interests concerning the result of the research but *less* legitimacy than claims made by a pluralist community that *also* prevents conflicts of interest. This is in line with Longino’s account of objectivity as gradual, but involves other strategies than those necessary for the fostering of pluralism of opinion. A potential disadvantage is the consequent loss of simplicity, in that the legitimacy of some influences cannot be judged in isolation. Social perspectives, for instance, can be illegitimate only in their context, i.e. if they reinforce a dominant and problematic pattern. On their own, they can be neither right, wrong, nor illegitimate. Contextualising scientific claims, therefore, adds another layer of complexity to the debate. Nevertheless, I hold that the work done within the values in science debate already covers and offers accounts of these various levels. Consequently, the framework we employ should do justice to the complexity of the interactions between science and society, too.

## Footnotes

1 Not all philosophers of science do so (Betz 2013; Hudson 2016; Hudson 2016; Mitchell 2004). But this paper is primarily addressing those who do.

2 I adopt the terms social legitimacy and authority from Holman and Wilholt (2022). Given that legitimacy, as well as authority, are concepts that are borrowed from the political domain, it is not always clear what exactly is meant by them in the context of science. They are also not the only concepts used to describe related phenomena. Other philosophers focus on integrity (Resnik & Elliott, 2019) or social responsibility (Kourany 2010). This paper does not attempt a clarification of the concepts of legitimacy and authority as such, but it tries to bring into view important aspects that can contribute to socially legitimate science in Holman and Wilholt’s sense.

3 Social perspective representation amounts here to what Hanna Pitkin (1972) has termed “descriptive representation”, that is representation based on similarity of demographic features.

4 Elizabeth Anderson describes the influence of evidence on our beliefs and values very eloquently in her 2004 paper on divorce research. She argues that it is exactly when experiences and evidence have no influence on our values (or opinions in Young’s terms) that they become dogmatic and problematic. Thanks to an anonymous reviewer for pointing me towards this text.

5 Scientists operating with the linear model hypothesised that there is a relation between foetal gonadal hormone exposure, for instance to testosterone, and later behavioural patterns such as homosexuality or gender role behaviour. Doell and Longino (1988) argued that even if there were a correspondence between the two phenomena this cannot be taken as proof for a causal relation because it treats intentional behaviour (e.g. the choice of erotic partners) as comparable to simple reflexes (observed in animal studies).

6 Interestingly, the fact that the Navy acted as patron did however indirectly also impact the ideological views that were represented within the community of oceanographers in the US. As Oreskes writes, scientists needed security clearance to work on classified military projects which excluded overtly critical or antimilitary voices (Oreskes, 2020, pp. 11, 486).

7 Although with slight differences, various philosophers of science agree that the replacing ideal for the value-free ideal should fulfil such dual function of guaranteeing epistemic adequacy and social legitimacy (cf. Kourany 2010, chapter 3; Kitcher 2001, 2011; Douglas 2016; Douglas 2009).

8 Arguably, the ideal should also prevent science and scientists from exerting an illegitimate influence on politics. I acknowledge that this reverse side of the science-society interactions is badly captured in the framework of this paper. I nevertheless consider it an improvement if it manages to capture the complexities of contextual influences on science better than the currently dominant values framework.

9 I treat social legitimacy as a necessary but not sufficient condition for scientific judgements to be authoritative. Epistemic criteria continue to be of central importance for science’s veracity, universality and authority. Authority can therefore not be reduced to social legitimacy and the management of contextual influences will get us closer but not suffice to demarcate good from bad science.

10 I presuppose here that the influence of opinions cannot be prevented all together. As mentioned at the outset of this paper, this assumption is not universally shared. For those who disagree at this level, my account will likely not be convincing.

11 A different approach would be to restrict the influence of opinions in specific phases of research. Douglas (2009) for instance proposes that societal values (in her terminology) should only play an indirect role in “internal phases” of research.

12 “Right values approach “ is a term that I take from Douglas (2016). Rolin (2021) speaks of “proper values”, Holman and Wilholt (2022) of “axiological demarcation strategies”.

13 Kitcher does not speak of interests only, he frequently uses the terms preferences, perspectives and values as well. Yet he presents his project as an attempt to bring different individual interests “under a single measure” (Kitcher, 2001, p. 115) and thereby determine the collective good.

14 Douglas makes similar appeals to involve stakeholders in a setting where power is distributed equally (Douglas, 2009, p. 165).

15 This has also been discussed by Kourany (2010, pp. 58–62) and Rolin (2016, 2021).
